# 2547 Long-term response to treatment and disease recurrence in a prospective cohort of morphea patients

**DOI:** 10.1017/cts.2018.170

**Published:** 2018-11-21

**Authors:** Stephanie Florez-Pollack, Jack C. O’Brien, Heidi T. Jacobe

**Affiliations:** University of Texas Southwestern Medical Center

## Abstract

OBJECTIVES/SPECIFIC AIMS: Morphea (localized scleroderma) is an autoimmune disease characterized that is widely thought to have a monophasic course, in which an initial period of inflammation (activity) ultimately results in scarring, atrophy, and functional impairment (damage). Understanding the long-term clinical course of morphea is important for the planning of future interventional studies, and as a tool for clinicians in determining risk for poor disease outcomes. METHODS/STUDY POPULATION: We conducted a prospective cohort study of 130 participants enrolled in the Morphea in Children and Adults Cohort over a median follow-up time of 4.3 years, to determine the rates of response to treatment and disease recurrence as measured by the Localized Scleroderma Cutaneous Assessment Tool (LoSCAT). To determine risk factors for recurrence of disease activity, survival analysis using the log-rank test was used to compare subgroups by morphea type, therapy, and age at disease onset. RESULTS/ANTICIPATED RESULTS: Within a 1-year follow-up period, 66% of patients treated with methotrexate and 46% of patients with UVA1 phototherapy had achieved complete response to treatment. In patients who had achieved response to treatment, 29% experienced disease recurrence at an average of 1.7 years after documented disease inactivity. Patients with generalized morphea experienced higher recurrence rates than those with linear morphea (HR: 3.03, 95% CI: 1.48–6.22), and those treated with UVA1 phototherapy had higher recurrence over those treated with methotrexate (HR: 2.33, 95% CI: 1.03–5.31). In patients with follow-up periods longer than 5 years (n=50), disease recurrence was observed in 44% of patients and the majority of recurrence represented new activity in an area of pre-existing morphea (82%). DISCUSSION/SIGNIFICANCE OF IMPACT: This study highlights the previously under-studied dynamic long-term course of morphea, and identifies the clinical characteristics that predispose patients to having a relapsing-remitting course. We conclude that patients with morphea should be followed closely over time even in the absence of disease activity due to the potential for disease recurrence.

